# Quality of Life and Mortality of Long-Term Colorectal Cancer Survivors in the Seattle Colorectal Cancer Family Registry

**DOI:** 10.1371/journal.pone.0156534

**Published:** 2016-06-02

**Authors:** Scott V. Adams, Rachel Ceballos, Polly A. Newcomb

**Affiliations:** Public Health Sciences Division, Fred Hutchinson Cancer Research Center, Seattle, WA, United States of America; Iranian Institute for Health Sciences Research, ACECR, ISLAMIC REPUBLIC OF IRAN

## Abstract

**Background and Aim:**

Because most colorectal cancer patients survive beyond five years, understanding quality of life among these long-term survivors is essential to providing comprehensive survivor care. We sought to identify personal characteristics associated with reported quality of life in colorectal cancer survivors, and sub-groups of survivors potentially vulnerable to very low quality of life.

**Methods:**

We assessed quality of life using the Veterans RAND 12-item Health Survey within a population-based sample of 1,021 colorectal cancer survivors in the Seattle Colorectal Cancer Family Registry, approximately 5 years post-diagnosis. In this case-only study, mean physical component summary scores and mental component summary scores were examined with linear regression. To identify survivors with substantially reduced ability to complete daily tasks, logistic regression was used to estimate odds ratios for “very low” summary scores, defined as a score in the lowest decile of the reference US population. All cases were followed for vital status following QoL assessment, and mortality was analyzed with Cox proportional hazards regression.

**Results:**

Lower mean physical component summary score was associated with older age, female sex, obesity, smoking, and diabetes or other co-morbidity; lower mean mental component summary score was associated with younger age and female sex. Higher odds of very low physical component summary score was associated with older age, obesity, less education, smoking, co-morbidities, and later stage at diagnosis; smoking was associated with higher odds of very low mental component summary score. A very low physical component score was associated with higher risk of mortality (hazard ratio (95% confidence interval): 3.97 (2.95–5.34)).

**Conclusions:**

Our results suggest that identifiable sub-groups of survivors are vulnerable to very low physical components of quality of life, decrements that may represent meaningful impairment in completing everyday tasks and are associated with higher risk of death.

## Introduction

A majority of patients with colorectal cancer (CRC) will survive five years or longer, and for those diagnosed with localized disease, 5-year survival exceeds 85%[1]. Thus a better understanding of quality of life (QoL) among survivors has become an essential component of providing comprehensive and directed survivor care. In particular, identification of survivors at highest risk of very low QoL may be important for developing appropriate and effective long-term survivorship plans.

Several studies of QoL in long-term (≥5 years) CRC survivors have been recently reviewed[2]. However, a limited number of these studies were population-based, investigated global measures of QoL, and included a large number of survivors [3–9]. The results of these studies suggest that, on average, long-term CRC survivors experience good QoL with only moderately lower physical functioning associated with older age, obesity, co-morbidities, smoking, and lower socioeconomic status [2, 7–10]. Psychological QoL has generally been observed to be comparable to the general population despite possibly higher depression scores[2, 3, 9–12].

In this study, we evaluated QoL in the Seattle Colorectal Cancer Family Registry (CCFR) [13]. Our aims were to identify personal characteristics of survivors associated with mean QoL among survivors in this large, population-based sample. In addition, the previously reported magnitudes of mean differences in QoL scores comparing groups of survivors were modest and may not reflect meaningful deficits in functioning.[14–18] Therefore, we investigated the distribution of QoL among survivors in order to identify sub-groups of CRC survivors potentially at higher risk of experiencing poor QoL associated with substantial impairment.

Finally, we investigated the extent to which QoL scores were associated with subsequent mortality. Prior studies of the relationship between health-related QoL and survival in CRC patients have suggested that QoL soon after diagnosis or early in treatment is prognostic independent of clinical characteristics; results suggest that QoL is an independent predictor of survival especially among patients with advanced CRC[19–25]. Much less has been reported regarding QoL and subsequent survival among long-term CRC survivors. In the general population, health-related QoL has also been associated with risk of mortality [26, 27] Therefore we explored the association of QoL with mortality among long-term CRC survivors.

## Materials and Methods

### Ethics statement

This study was approved by the Fred Hutchinson Cancer Research Center institutional review board, and conducted consistent with the 1964 Helsinki declaration and amendments. All participants provided written informed consent.

### Study population

The Seattle CCFR is a population-based registry that includes primary invasive colorectal cancer cases diagnosed through Puget Sound Region Surveillance, Epidemiology, and End Results (SEER) cancer registry, and their first degree relatives. Details of the registry enrollment protocols, eligibility criteria, and response rates have been published[13, 28, 29]. Cases diagnosed 1998–2004 were typically enrolled within 8 months of diagnosis, and response rates averaged 75% among eligible cases alive at contact[28, 29].

### Baseline data collection

Cases completed standardized telephone interviews covering CRC risk factors, including demographics, height and weight (2 years prior to baseline interview), smoking history (2 years prior to baseline interview), lifestyle and dietary factors, medications, cancer screening procedures, and history of digestive diseases (colitis, Crohn’s disease, irritable bowel syndrome, or diverticulitis) and diabetes. Body mass index (BMI, kg/m^2^) was calculated as weight divided by height squared. Data on stage at diagnosis and tumor site were obtained through the Puget Sound SEER cancer registry.

### Follow-up data collection including quality of life (QoL)

Cases still alive approximately five years after diagnosis were re-contacted for follow-up telephone interviews (90%) or mailed questionnaires between 2004 and 2012. The mean time between baseline data collection and follow-up data collection was 5.5 years (interquartile range: 5.1 to 5.8 years).

Follow-up interviews covered cancer surveillance tests, incident cancers, medication use, and updates to family history of cancer since the baseline interview. QoL was assessed with the Veterans RAND 12-item Health Survey (VR-12), a standard and validated survey tool used to assess health-related physical and mental QoL and which is available in the public domain [14, 30]. The VR-12 has been validated in populations other than veterans, including Medicare enrollees[14]. The VR-12 was adapted from the copyright-protected 12-item Short-Form Health Survey (SF-12) [15, 31]. The physical component summary (PCS) score and mental component summary (MCS) score were each calculated from 6 questions following published procedures; in this method, PCS and MCS scores are scaled for comparison to a normative population[14, 30].[32][15]

Cases were followed for vital status through linkage to the National Death Index through the Puget Sound SEER cancer registry, from enrollment in the SCCFR to July 1, 2013.

### Study participation rates

The population from which sample for this study was drawn included CRC cases enrolled 1998–2004 (N = 2,106 total). Of these cases, 1,533 were alive 5 years post-diagnosis and therefore eligible for follow-up; 1,095 (71%) responded to the follow-up questionnaire. We subsequently excluded cases with incomplete VR-12 or covariate data (N = 74 cases). Hence, in total, 1,021 survivors completed baseline and follow-up questionnaires including VR-12 components necessary for calculation of MCS and PCS scores, and had complete information on analytical variables, and were included in these analyses.

Compared to the 1,021 survivors included in the present analyses, the non-participating 1,011 CRC cases (“non-responders”, including deceased cases) were approximately the same age at diagnosis (mean 58.6 years and 58.2 years), and a larger proportion of non-responders were male (58% to 52%), smokers (14% to 10%), and diagnosed with distant CRC (15% to 3%).

### Statistical analysis

Unadjusted arithmetic mean PCS and MCS scores were calculated for survivors. We also constructed histograms to visually qualitatively examine the distribution of PCS and MCS scores in survivors.

Multivariate linear regression was used to estimate the association of mean PCS and MCS scores with personal characteristics. The significance of each personal characteristic in the model was assessed with a two-tailed Wald test (significance at P<0.05). For presentation, coefficients of the linear regression model are expressed as the difference in mean PCS or MCS score associated with each category compared to the reference category. The intercept of the linear regression model is the mean PCS or MCS predicted for (hypothetical) persons in the referent category of all variables.

In a separate analytical approach, to identify survivors with meaningfully lower QoL scores[15, 17], participants’ PCS and MCS scores were compared to the lowest decile in the normative score distribution for the general US population, *i*.*e*., a normal distribution with mean 50 and standard deviation 10 [15, 32]. Thus, PCS and MCS scores in survivors and relatives were categorized as either below, or equal to or above, the 10^th^ percentile cut-off (<37.2 or ≥37.2 respectively) previously established for the US general population. Hence, scores less than 37.2 were labeled “very low.” Multivariate logistic regression was then used to estimate odds ratios (ORs) and 95% confidence intervals (95% CIs) for a PCS or MCS scores by category of personal characteristics. Therefore, in this case-only study, there were no controls; instead the normed QoL scores from the general population are used as a reference to identify cases with objectively very low QoL scores[15]. To examine sensitivity to the choice of the 10^th^ percentile as the cut-off for “very low” PCS or MCS, in additional analyses the cut-off was changed to the lowest quartile (<43.3) or lowest 5^th^ percentile (<33.6).

For statistical analysis of survival, time was measured in days from completion of the follow-up questionnaire assessing QoL. Kaplan-Meier product limits of overall survival probability were calculated comparing survivors according to PCS and MCS scores and compared with a global log-rank test. Subsequently, Cox proportional hazards regression was used to estimate hazard ratios (HRs) of death associated with very low PCS or MCS score (<37.2), compared to survivors with scores ≥37.2 (i.e., not “very low”). HR estimates for PCS were adjusted for MCS and estimates for MCS were adjusted for PCS, and additionally adjusted for age and sex and stratified by stage at diagnosis. In a second model, HRs for death was estimated comparing survivors in each stratum of one or both PCS and MCS scores <37.2 to survivors with both PCS and MCS ≥37.2. Additional adjustment for smoking, BMI, marital status, education, race and/or diabetes did not materially change the results of analysis.

Statistical analyses were completed using Stata/SE v14 (StataCorp, College Station, TX).

## Results

Long-term CRC survivors in this study were typically over 50 years old at diagnosis, and the numbers of men and women were similar (Table 1). A majority of CRC survivors were either overweight or obese, had some history of smoking cigarettes, were married, and reported some post-secondary education. The majority of survivors who were alive and participated in the 5 year follow-up interview were diagnosed with local stage CRC, and were more likely to have had colon cancer than rectal cancer.

**Table 1:** Association of personal characteristics at enrollment with mean Physical Component Summary (PCS) and Mental Component Summary (MCS) scores from the VR-12, for survivors at 5 year follow-up in the Seattle Colorectal Cancer Family Registry.

	N (%)	PCS			MCS		
		Difference in Mean PCS	(95%CI)	P	Difference in Mean MCS	(95%CI)	P
Age (y)^a^							
<50	228 (22)	ref.		<0.001	ref		<0.001
50–59	272 (27)	-1.5	(-3.4, 0.3)		2.6	(1.1, 4.1)	
60–69	337 (33)	-3.1	(-5.0, -1.2)		4.5	(3.0, 6.0)	
≥70	184 (18)	-5.9	(-8.0, -3.7)		3.7	(1.9,5.4)	
Sex				0.001			0.001
Male	530 (52)	ref			ref		
Female	491 (48)	-2.3	(-3.6, -0.9)		-1.7	(-2.8, -0.7)	
BMI (kg/m^2^)^b^				<0.001			0.70
<25	335 (33)	ref			ref		
25–29.9	408 (40)	-0.9	(-2.4, 0.6)		-0.3	(-1.5, 0.9)	
≥30	278 (27)	-3.7	(-5.6, -1.9)		0.2	(-1.2, 1.6)	
Marital Status^a^				0.49			0.66
Not married	259 (25)	-0.5	(-2.1, 1.0)		-0.3	(-1.5, 1.0)	
Married or living as married	762 (75)	ref			ref		
Race				0.22			0.73
White	933 (91)	ref			-0.3	(-2.2, 1.6)	
Other	88 (9)	1.3	(-0.8, 3.4)		ref		
Education^a^				<0.001			0.19
High school or less	318 (31)	-1.5	(-3.2, 0.3)		-0.6	(-1.9, 0.8)	
Some college or technical education	335 (33)	ref			ref		
College graduate	368 (36)	2.4	(0.8, 4.0)		0.6	(-0.6, 1.7)	
Cigarette Smoking^a^				<0.001			0.11
Never	414 (41)	ref			ref		
Former	502 (49)	-1.5	(-2.9, -0.1)		-0.3	(-1.4, 0.8)	
Current	105 (10)	-5.5	(-8.1, -2.9)		-2.3	(-4.5, 0.2)	
Colorectal Co-morbidities^a^,^c^				0.049			0.16
None	837 (82)	ref			ref		
One or more	184 (18)	-1.8	(-3.5, 0.0)		-1.0	(-2.4, 0.4)	
Diabetes^a^,^d^				<0.001			0.18
No	899 (88)	ref			ref		
Yes	122 (12)	-5.0	(-7.4, -2.6)		-1.2	(-2.9, 0.6)	
Stage at Diagnosis				0.12			0.57
Local	517 (51)	ref			ref		
Regional	454 (44)	-0.5	(-1.9, 0.9)		-0.8	(-1.9, 0.2)	
Distant	30 (3)	-1.1	(-5.7, 3.6)		1.9	(-0.2, 4.1)	
Unstaged or unknown	20 (2)	-6.3	(-11.6, -1.1)		-0.8	(-4.7, 3.1)	
Site of Tumor				0.59			0.74
Colon	619 (61)	ref			ref		
Rectum	349 (34)	-0.7	(-2.1, 0.7)		0.2	(-0.9, 1.3)	
Unknown	53 (5)	-0.8	(-3.9, 2.3)		-0.7	(-2.9, 1.6)	
Intercept ^e^		52.5	(50.4, 54.8)		53.2	(51.4, 55.1)	

^a^ At Colon Cancer Family Registry enrollment.

^b^ Two years prior to baseline interview.

^c^ Self-report of physician diagnosis of colitis, Crohns disease, diverticulitis, or irritable bowel syndrome.

^d^ Self-report of physician diagnosis of diabetes.

^e^ The intercept from the linear regression model is the mean PCS and MCS among (hypothetical) individuals in the referent category for all variables.

The unadjusted mean (95% CI) PCS score was 45.1 (44.4–45.8) among survivors, slightly lower than the US general population means previously reported for all adults (mean 50) and for persons age>45 (mean 46.3). Lower adjusted mean PCS score was associated with older age, female sex, higher BMI, lower educational attainment, and smoking history (Table 1). In the CRC survivors in the present study, digestive co-morbidities and self-report of physician diagnosis of diabetes were also associated with lower PCS. Overall, stage at diagnosis was not significantly associated with mean PCS although mean PCS was lower among the few unstaged survivors.

Unadjusted mean (95% CI) MCS score was 54.1 (53.6–54.6) among survivors, somewhat higher than the US general population of all adults (mean 50) and those >45 years old (mean 51.2)[33]. Among the CRC survivors in our sample, higher adjusted mean MCS was associated with older age and male sex (Table 1).

**Fig 1:**
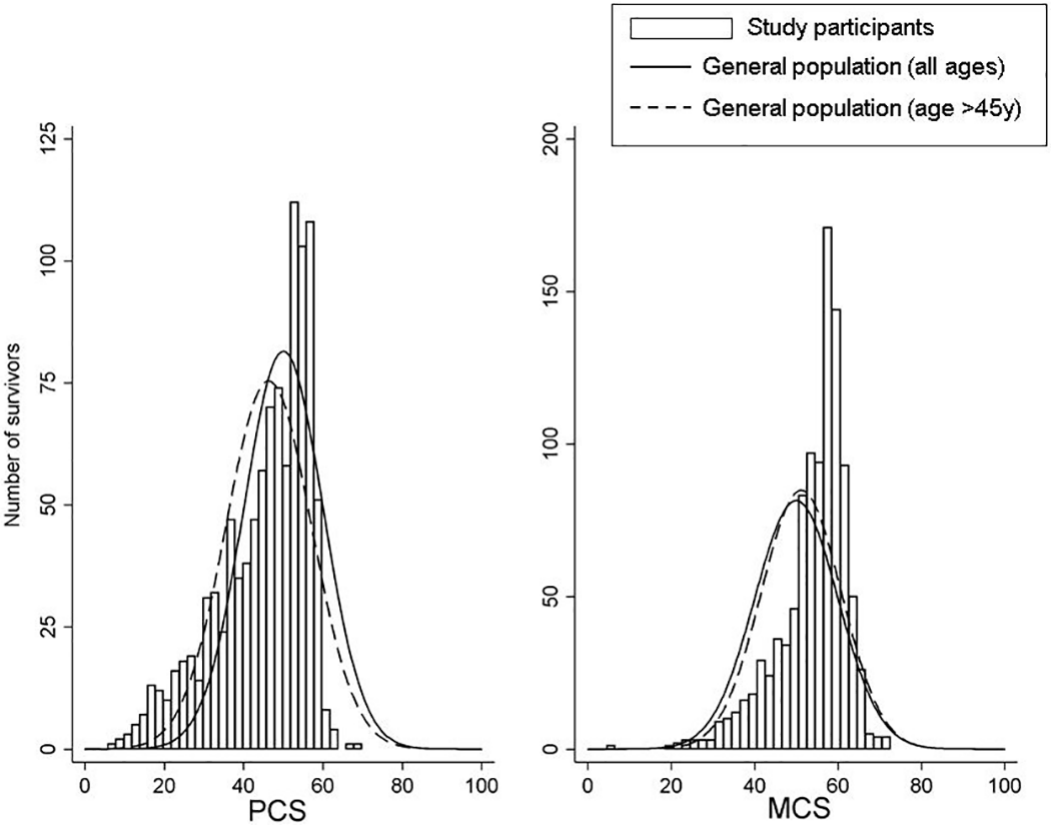
Distribution of Physical Component Summary (PCS) and Mental Component Summary (MCS) scores among survivors (black bars; bin width is 2 units), and normative reference populations. Solid line represents the US general population, with normal distribution with mean (standard deviation) of 50 (10). Dashed line represents the US population over age 45 years, with mean PCS 46.3 (10.8) and mean MCS 51.1 (9.6)[14, 15, 33]. Curves have been scaled such that the area under each is equal to the number of participants, 1,021, and the y-axis is the number of survivors in each bar.

The histogram of PCS scores showed a distinct heavy tail at low values among survivors that was visually distinguishable from the normative curve for the general population and the population over 45 years old (Fig 1). MCS score distributions did not show a similar tail at low values, but did evidence a larger proportion of survivors above comparative population means. Quantitatively, 23% and 5% of survivors had very low (below the value of the lowest decile in the reference population, 37.2) PCS and MCS, respectively.

Logistic regression results showed that older age, obesity, lower educational attainment, smoking, and co-morbidities or diabetes were associated with higher odds of very low PCS (Table 2). Regional, distant or “unstaged or unknown” stage were progressively associated with higher odds of very low PCS. Higher odds of very low MCS were significantly associated only with smoking, and this association was most pronounced for “current smokers” at enrollment.

**Table 2:** Associations of survivor personal characteristics with VR-12 Physical Component Summary (PCS) or Mental Component Summary (MCS) score below 10^th^ percentile value in the general population, among CRC survivors approximately 5 years after diagnosis, Seattle Colon Cancer Family Registry (enrolled 1998–2004).

	PCS below 10^th^ percentile (<37.2)			MCS below 10^th^ percentile (<37.2)		
	OR^a^	(95% CI)	P^b^	OR^a^	(95% CI)	P^b^
Age (y)^c^			0.001			0.13
<50	ref			ref		
50–59	1.34	(0.82, 2.21)		0.37	(0.15, 0.92)	
60–69	1.52	(0.95, 2.43)		0.52	(0.25, 1.10)	
≥70	2.74	(1.63, 4.62)		0.69	(0.29, 1.65)	
Sex			0.12			0.19
Male	ref			ref		
Female	1.29	(0.93, 1.78)		1.48	(0.82, 2.67)	
BMI (kg/m^2^)^d^			0.014			0.69
<25	ref			ref		
25–29.9	1.02	(0.69, 1.50)		1.13	(0.59, 2.16)	
≥30	1.81	(1.20, 2.74)		0.80	(0.34, 1.85)	
Marital Status^c^			0.94			0.60
Not married	0.99	(0.69, 1.41)		0.83	(0.41, 1.66)	
Married or living as married	ref			ref		
Race			0.51			0.25
White	ref					
Other	0.83	(0.47, 1.46)		1.71	(0.69, 4.25)	
Education^c^			<0.001			0.35
High school or less	1.58	(1.10, 2.28)		1.02	(0.54, 1.93)	
Some college or technical education	ref			ref		
College graduate	0.64	(0.43, 0.97)		0.60	(0.28, 1.29)	
Cigarette Smoking^d^			<0.001			0.04
Never	ref			ref		
Former	1.37	(0.97, 1.96)		1.60	(0.78, 3.32)	
Current	2.79	(1.65, 4.75)		3.06	(1.29, 7.23)	
Colorectal Co-morbidities^c^,^e^			0.005			0.30
None	ref			ref		
One or more	1.75	(1.19, 2.58)		1.46	(0.72, 2.99)	
Diabetes^c^,^f^			0.013			0.46
No	ref					
Yes	1.81	(1.13, 2.88)		1.40	(0.58, 3.37)	
Stage at Diagnosis			0.01			0.19
Local	ref			ref		
Regional	1.22	(0.88, 1.70)		1.14	(0.63, 2.06)	
Distant	2.61	(1.08, 6.30)		NE^g^		
Unstaged or unknown	3.72	(1.33, 10.39)		1.18	(0.14, 10.01)	
Site of Tumor			0.45			0.80
Colon	ref			ref		
Rectum	1.17	(0.83, 1.66)		1.20	(0.66, 2.20)	
Unknown	1.41	(0.73, 2.71)		1.00	(0.30, 3.29)	

^a^ Adjusted for all variables listed.

^b^ P value of joint Wald test of categorical coefficients in the logistic regression model.

^c^ At Colon Cancer Family Registry enrollment.

^d^ Two years prior to baseline interview.

^e^ Self-report of physician diagnosis of colitis, Crohns disease, diverticulitis, or irritable bowel syndrome.

^f^ Self-report of physician diagnosis of diabetes.

^g^ Not estimated (NE); no survivors diagnosed with distant stage reported MCS below the 10^th^ percentile.

Changing the cut-off for very low PCS and MCS to the 5^th^ or 25^th^ percentile values had no substantial impact on the interpretation of results.

**Fig 2:**
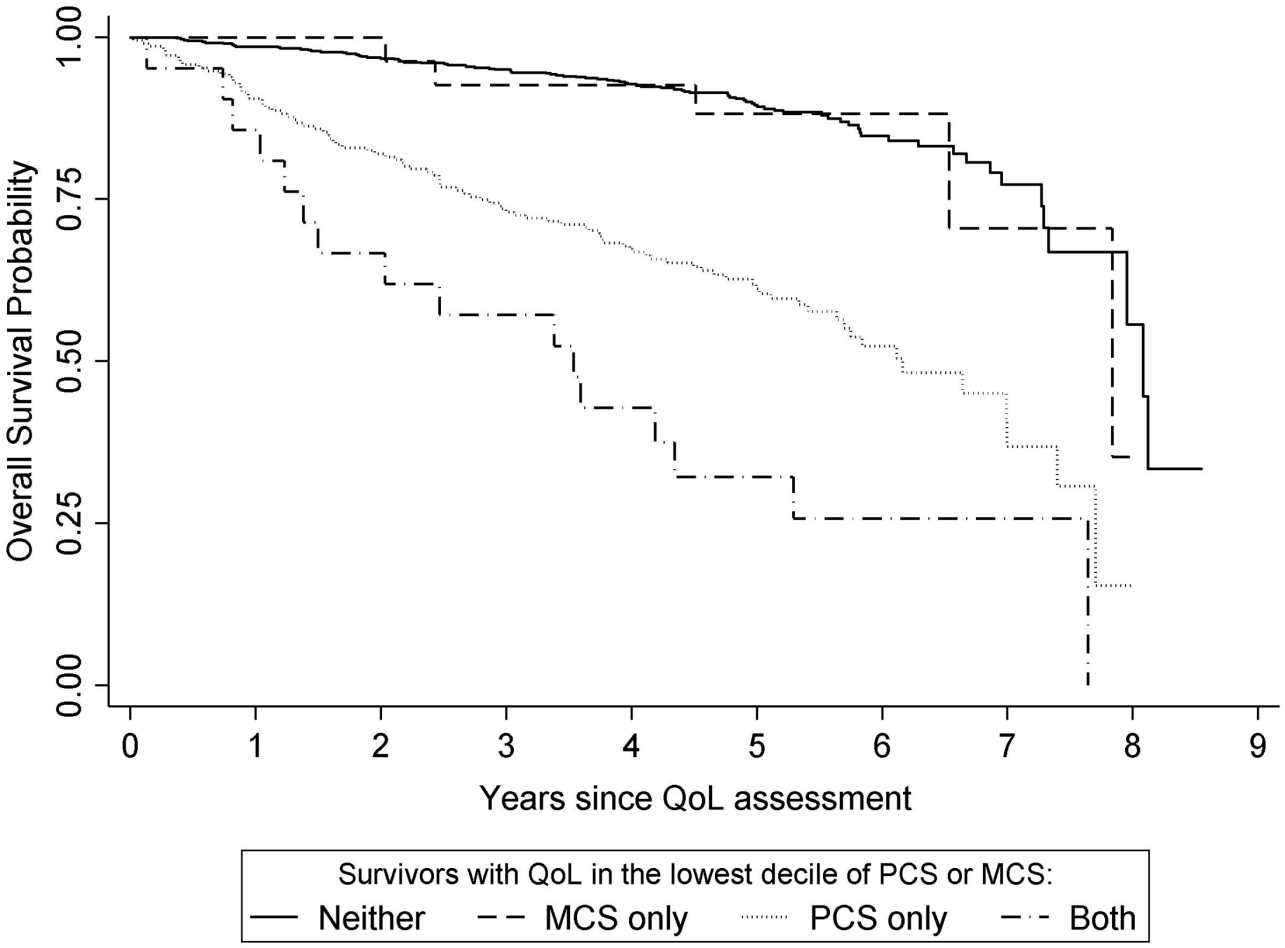
Kaplan-Meier survival curves comparing survivors by Physical Component Summary (PCS) and Mental Component Summary (MCS) scores.

During a median of 4.8 years follow-up after completion of the QoL survey (at ≥5 years post-diagnosis), 204 deaths occurred, 59 of which were due to CRC. The mean mortality rate was 44.6 (95% CI: 38.9–51.1) deaths per 1,000 person-years. Differences in overall survival between survivors with PCS or MCS scores <37.2 and survivors with PCS or MCS scores ≥37.2 were evident (Fig 2; log-rank P< 0.0001). A very low PCS score was associated with higher hazard of death (HR (95% CI): 3.97 (2.95, 5.34)), adjusted for MCS score (Table 3), and a very low MCS score was also associated with higher hazard of death (HR (95% CI): 1.98 (1.19, 3.28)) adjusted for PCS score. However, MCS was not significantly associated with mortality among survivors with PCS score above the lowest decile; in contrast, very low PCS was associated with higher risk of death among survivors in either stratum of MCS score (Fig 2 and Table 3).

**Table 3:** Association of subsequent survival with quality of life Physical Component Summary (PCS) and Mental Component Summary (MCS) score assessed approximately 5 years after colorectal cancer diagnosis.

PCS		deaths / N	HR^a^,^b^	95% CI
≥10^th^ %tile		94 / 783	ref.	
<10^th^ %tile		110 / 238	3.97	(2.95, 5.34)
per 10 points lower		204 / 1021	1.71	(1.53, 1.91)
MCS		deaths / N	HR^a^,^b^	95% CI
≥10^th^ %tile		183 / 970	ref.	
<10^th^ %tile		21 / 51	1.98	(1.19, 3.28)
per 10 points lower		204 / 1021	1.39	(1.20, 1.62)
PCS	MCS	deaths / N	HR^a^	95% CI
≥10^th^ %tile	≥10^th^ %tile	89 / 753	ref.	
≥10^th^ %tile	<10^th^ %tile	5 / 30	1.67	(0.78, 3.58)
<10^th^ %tile	≥10^th^ %tile	94 / 217	3.90	(2.87, 5.30)
<10^th^ %tile	<10^th^ %tile	16 / 21	8.24	(4.26, 15.96)

^a^ Adjusted for age and sex, and stratified by stage.

^b^ HR for PCS adjusted for MCS category, HR for MCS adjusted for PCS category.

## Discussion

In this study, unadjusted average physical QoL was modestly lower, and average mental QoL somewhat higher, than reference values for the general US population [15, 33]. Although an imperfect comparison, the difference in mean PCS score between CRC survivors and the population over 45 years old was approximately 1.2 points, an absolute difference on the VR-12 QoL scale that probably does not represent a meaningfully lower ability to complete daily tasks, and is unlikely to be clinically important[14, 15, 34]. Similarly, the difference of 3 points in mean MCS between survivors and the older general population may not be large enough to be important. Because earlier studies that have demonstrated lower QoL among patients in the year or so following CRC diagnosis[35, 36], our study provides some suggestive evidence that, on average, CRC patients who survive approximately 5 years from diagnosis can expect to return to quality of life typical for their age, in spite of possible continuing challenges stemming from CRC.

Visual inspection of the distribution of PCS and MCS scores among CRC survivors revealed a larger than expected sub-group of survivors with low PCS score. The lowest decile of PCS scores in the general population represents a PCS of 13 or more points below the general population mean of 50, or approximately 9 or more points below the mean PCS for the population over age 45, deficits large enough to reflect a meaningfully lower ability to complete daily tasks and usual activities[14, 15, 17]. We sought to characterize CRC survivors in this group with very low PCS scores. Our results showed that persons diagnosed at an older age, with higher BMI, less education, a history of smoking, and co-morbidities including diabetes may be particularly vulnerable to experiencing low physical QoL. The underlying reasons for lower physical QoL may not be specific to CRC survivorship. Our results nonetheless suggest sub-groups of CRC survivors that may be at higher risk of lingering physical disabilities that interfere with everyday life, and which previously published reports have associated with a higher risk of mortality[16, 17]. Thus continued survivorship support may be especially warranted in these groups.

In contrast to the physical domain of QoL, in the mental domain, CRC survivors qualitatively showed modestly higher QoL than the general population with little evidence of a tail at low values. We identified only cigarette smokers as a risk factor for very low mental QoL. These findings suggest the possibility of “benefit finding” among CRC survivors, as has been observed previously in survivors of disparate cancers including CRC[37, 38].

The characteristics of groups of survivors identified to have lower mean QoL scores, and those with higher odds of very low QoL score, were similar. The association of co-morbidities with odds of very low PCS score was somewhat stronger than might have been expected based on the modest association of co-morbidities with mean PCS score.

Our results regarding mean QoL, and factors associated with differences in mean QoL scores in both mental and physical dimensions, in long-term CRC survivors are largely consistent with previous reports from other studies[2–4, 6, 11]. However, most earlier studies have generally focused on the mean scores, with a few exceptions[7, 8]. Therefore our additional highlighting of the disproportionate number of survivors with “very low” PCS scores represents an under-utilized approach.

Previous reports have found that QoL assessed soon after CRC diagnosis was independently related to survival [19–25]. In our study of long-term CRC survivors, we observed a strong association between QoL, particularly physical QoL score, and risk of mortality. Interestingly, the associations of very low QoL with mortality that we found among CRC survivors were very similar to reports examining the association of QoL with mortality in general population populations [26] and among individuals of comparable age to the CRC survivors in our study years[27, 39–41]. Thus our results suggest that as in the general population, very low QoL identifies long-term CRC survivors at higher risk of death. However, the absolute mortality rate of long-term CRC survivors in the present study was 44.6 deaths per 1,000 years, which was likely higher than the rate in the US general population of comparable age (e.g., approximately 23 and 36 per 1,000 person-years for ages 70–74 years and 75–79 years, respectively [42]), even though the majority of deaths among our study participants were due to causes other than CRC. More research would be necessary to more fully understand differences in mortality between long-term CRC survivors and the general population of comparable age.

Some limitations of our study should be considered in interpreting our results. These include our use of the VR-12, rather than a longer 36-item survey such as the VR-36 or Short Form 36 (SF-36), although given the sample size of our study, the VR-12 has been shown to be adequate to assess the two summary measures of QoL[14, 15, 31]. By using only summary scores from the broadly applicable VR-12, rather than a disease-specific QoL survey, we did not capture in detail specific deficits such as bowel problems. Our study only included survivors who lived at least ~5 years post-diagnosis, but our objective was to examine long-term survivors. However, CRC patients who were alive at the time of the scheduled 5 year follow-up interview but did not respond may have experienced lower QoL. An additional limitation is our lack of detailed data on CRC treatment, but we did include information on stage at diagnosis, and tumor site, factors that are important in determining treatment received. We were also unable to adjust for other potential but unmeasured confounders such as financial status and social support. Finally, reflecting the population underlying the Seattle CCFR, our results included modest numbers of racial or ethnic minorities, and therefore further research is needed to generalize to sub-populations within the US.

In summary, our results show that approximately 5 years after diagnosis, on average, long-term CRC survivors QoL approaches the status of the general population. Nonetheless, some CRC survivors, particularly those with co-morbidities, obese survivors, smokers, older survivors, and survivors with less education, experience very low physical QoL and continued long-term support may be needed to mitigate the association of low QoL with higher risk of death. These important themes should be conveyed in communications with CRC patients, their families, and caregivers.
